# Safety and feasibility of in-hospital autonomous transportation using a driverless mobility for patients with musculoskeletal disorders: preliminary clinical study to achieve mobility as a service in medical care

**DOI:** 10.1186/s12891-024-07417-x

**Published:** 2024-05-03

**Authors:** Hiroshi Takahashi, Kenji Suzuki, Tomofumi Nishino, Yosuke Shibao, Hiroshi Noguchi, Akihiro Kanamori, Tomokazu Yoshioka, Naoya Kikuchi, Daisuke Nozawa, Hajime Mishima, Masao Koda, Masashi Yamazaki

**Affiliations:** 1 Department of Orthopaedic Surgery, Institute of Medicine, University of Tsukuba, 1-1-1, Tennodai, Tsukuba City, Ibaraki 305-8575 Japan; 2 Center for Cybernics Research, University of Tsukuba, 1-1-1, Tennodai, Tsukuba City, Ibaraki 305-8575 Japan; 3 Department of Orthopaedic Surgery, Ibaraki Western Medical Center, 555, Otsuka, Chikusei City, Ibaraki 308-0813 Japan; 4 Department of Orthopaedic Surgery, Kikkoman General Hospital, 100, Miyazaki, Noda City, Chiba 278-0005 Japan

**Keywords:** In-hospital autonomous transportation, Driverless mobility, Musculoskeletal disorders, Mobility as a service

## Abstract

**Background:**

Recent advancements in and the proliferation of autonomous mobility technology, such as intelligent wheelchairs, have made it possible to provide mobility services for patients with reduced mobility due to musculoskeletal disorders. In the present study, we conducted a preliminary clinical study to assess the safety and feasibility of in-hospital autonomous transportation using a driverless mobility (wheelchair) for patients with musculoskeletal disorders.

**Methods:**

From January to February 2022, 51 patients with musculoskeletal disorders exhibiting gait disturbance who presented to our institution were included in the present study. Driverless mobility rides were conducted over a straight-line distance of 100 m from the orthopaedic outpatient reception to the payment counter after the outpatient consultation. We assessed the quality of life using an EQ-5D-5 L index and pain using a VAS score before riding the mobility to investigate the patient’s condition. After the ride, a questionnaire survey was conducted to assess patient satisfaction on a 5-point scale. In addition, adverse events during the mobility ride were investigated.

**Results:**

Overall satisfaction levels showed that 44 out of 51 (86%) patients rated the level as 3 or higher. There were no significant differences in the level of satisfaction based on the cause of disorders or EQ-5D-5 L Index. Among 19 patients who rated the level of satisfaction as 2–3, the ratio of postoperative patients and those with pain tended to be higher (*p* < 0.05). While 26 of 51 (51%) patients reported moments of feeling unsafe during the mobility ride, no actual adverse events, such as collisions, were observed.

**Conclusions:**

An in-hospital autonomous transportation service using a driverless mobility for patients with musculoskeletal disorders demonstrated high satisfaction levels and was safe with no severe adverse events observed. The expansion of autonomous mobility deployment is expected to achieve mobility as a service in medical care.

## Background

Degenerative musculoskeletal disorders, such as lumbar spinal stenosis, compression myelopathy, osteoarthritis, and osteoporosis, are significant public health problems for the elderly population which affect the quality of life (QOL) through reduced mobility due to severe pain, paralysis, and sarcopenia [1–3]. In recent years, due to an aging society, the number of patients with reduced mobility due to musculoskeletal disorders, particularly locomotive organ disorders, has been increasing. The recent report indicated that the incidence of locomotive syndrome stage 1 is up to 69.8% in the Japanese population [4]. In patients with reduced mobility due to locomotive organ disorders, osteoporotic pathological fractures by minor trauma such as falls are sometimes a problem [5]. Thus, when patients with reduced mobility due to musculoskeletal disorders visit hospitals, using various mobility services becomes necessary to avoid in-hospital falls. Generally, wheelchairs are often used for in-hospital mobility. However, several problems exist, such as physical strain on healthcare providers during patient transfers [6]. In recent years, there have been reports regarding intelligent wheelchairs. However, there are problems related to patient learning curves and adaptability [7]. The development and proliferation of autonomous mobility technology in driverless electric wheelchairs in recent years, facilitated by advancements in sensors and mapping technologies, has made it possible to provide mobility services for patients with reduced mobility due to musculoskeletal disorders and who have difficulty going to their destinations independently [8]. However, to our knowledge, no reports have demonstrated and evaluated the patient-related outcomes of in-hospital autonomous patient transportation using a driverless wheelchair. In the present study, we conducted a preliminary clinical study to assess the safety and feasibility of in-hospital autonomous transportation services using a driverless mobility (wheelchair) for patients with reduced mobility due to musculoskeletal disorders.

## Methods

### Patient population

The present study was approved by the Institutional Review Board of our institution. Informed consent for participation in the study and use of their data was obtained from all the patients. The present study was conducted from January 17 to February 14, 2022. The operating hours of the mobility were from 10:00 to 12:00 and from 14:00 to 16:00 daily. Patients who could barely walk and had a gait disturbance due to severe pain, paralysis, or sarcopenia were included in the present study. Patients who could walk alone with upper extremity disorders or who were unable to walk at all and regularly used wheelchairs were excluded. Patients who did not wish to ride the wheelchair were also excluded. Ultimately, 51 patients with reduced mobility due to musculoskeletal disorders were included in the present study.

### Use of driverless mobility (wheelchair)

**Fig. 1:**
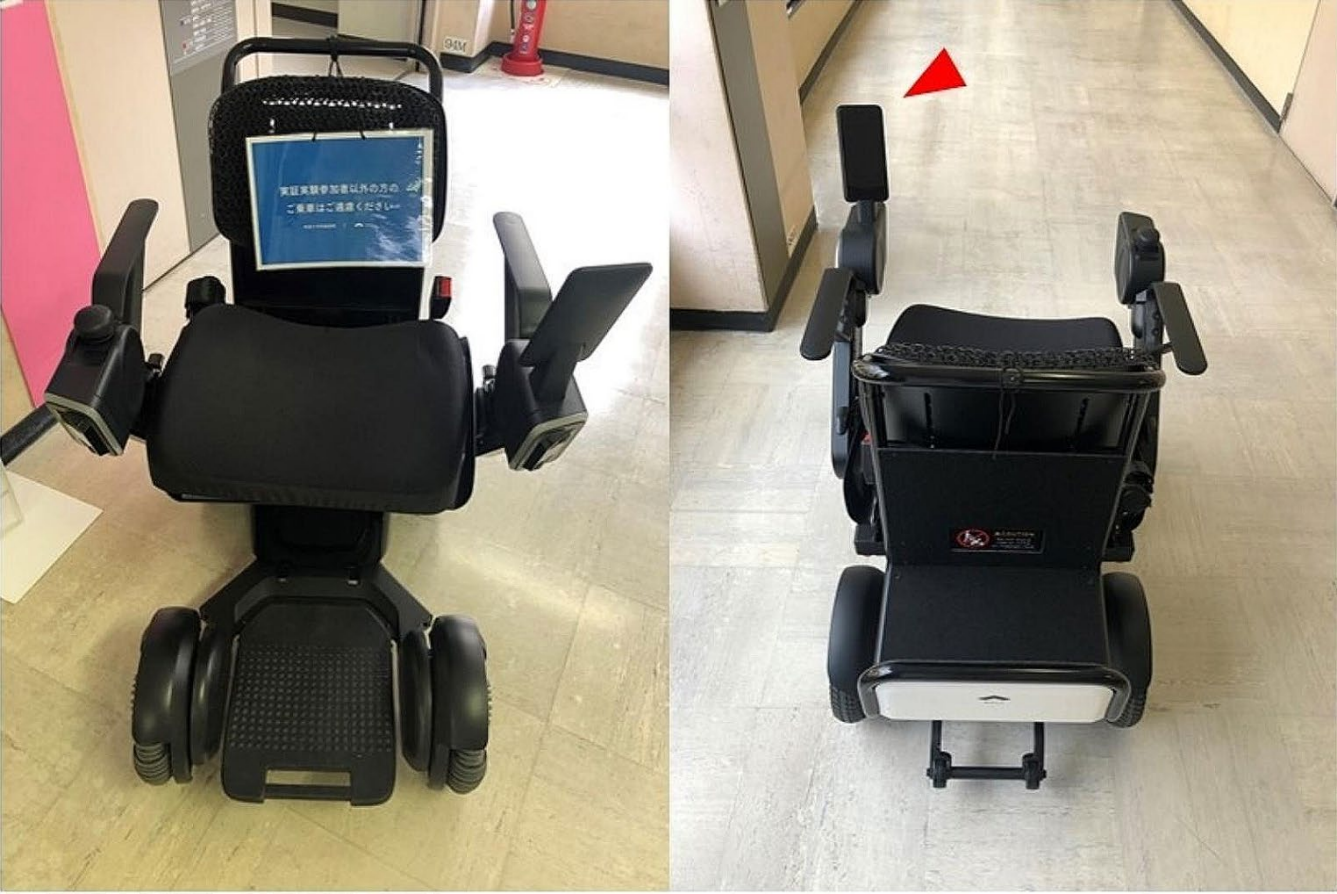
The driverless mobility used in the present study is manufactured by WHILL Inc. The arrowhead indicates the tablet device for the user

In the present study, the driverless mobility system manufactured by WHILL Inc. was used (Fig. 1). In this system, the wheelchair autonomously drives from a starting point to a predetermined destination by storing map information. The occupant can use this system to move automatically to the destination by setting a tablet device at the time of departure. The wheelchair operates by analyzing the surrounding conditions detected by sensors to avoid collisions. After the patient arrives at the destination, the wheelchair autonomously returns to the starting point.

### Setting the demonstration area

**Fig. 2:**
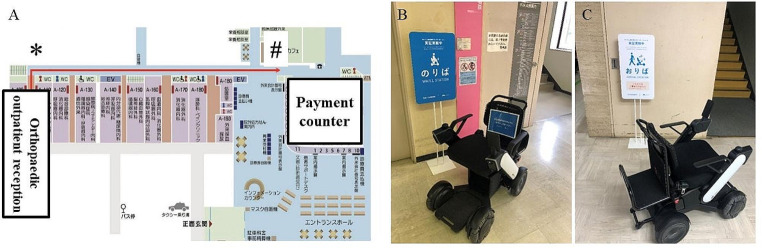
**(A)** The map of our hospital. The starting point (Orthopaedic outpatient reception) is indicated by an asterisk. A hash or pound symbol indicates the destination (near the payment counter). **(B)** The starting point. **(C)** The destination

Driverless mobility rides were conducted over a straight-line distance of 100 m from the orthopaedic outpatient reception to the payment counter after the outpatient consultation (Fig. 2). Before the start of the trial, the driverless mobility was programmed with map information. In addition, to verify the safety of mobility riding, multiple cameras were installed in the hospital corridors (ceiling area) to capture interactions with and overtaking of the other patients during the demonstration.

### Evaluation of patient background and outcome measures

The EuroQol 5 dimensions 5-level (EQ-5D-5 L) index was used to assess the patient QOL, and a Visual Analog Scale (VAS; 100 mm) score was used to assess the patient pain state before riding the mobility. After the ride, a questionnaire survey was conducted to assess the satisfaction and the riding comfort on a 5-point scale, and the presence of perceived dangers in the scenes. The EQ-5D-5 L index was obtained using the Japanese version of the EQ-5D-5 L value, which is estimated using the crosswalk methodology developed by the EuroQol Group [9, 10]. We divided the patients into two groups: a satisfaction group (satisfaction rating over 4; group S) and a dissatisfaction group (rating under 3; group D), and analyzed the difference in EQ-5D-5 L index and VAS scores between the two groups. In addition, the occurrences of adverse events during the mobility ride, such as collisions, were investigated using a preinstalled camera.

### Statistical analyses

The results are presented as the mean ± standard deviation. A Mann–Whitney *U* test was used to compare the relationship between satisfaction and EQ-5D domains. A Student *t-*test was used to determine the relationship between the satisfaction and pain VAS scores. *P* < 0.05 was considered significant in the tests of statistical inference. All statistical analyses were performed using the JMP software package (version 14.2.0; SAS Institute, Cary, NC, USA).

## Results

### Patient characteristics

**Fig. 3:**
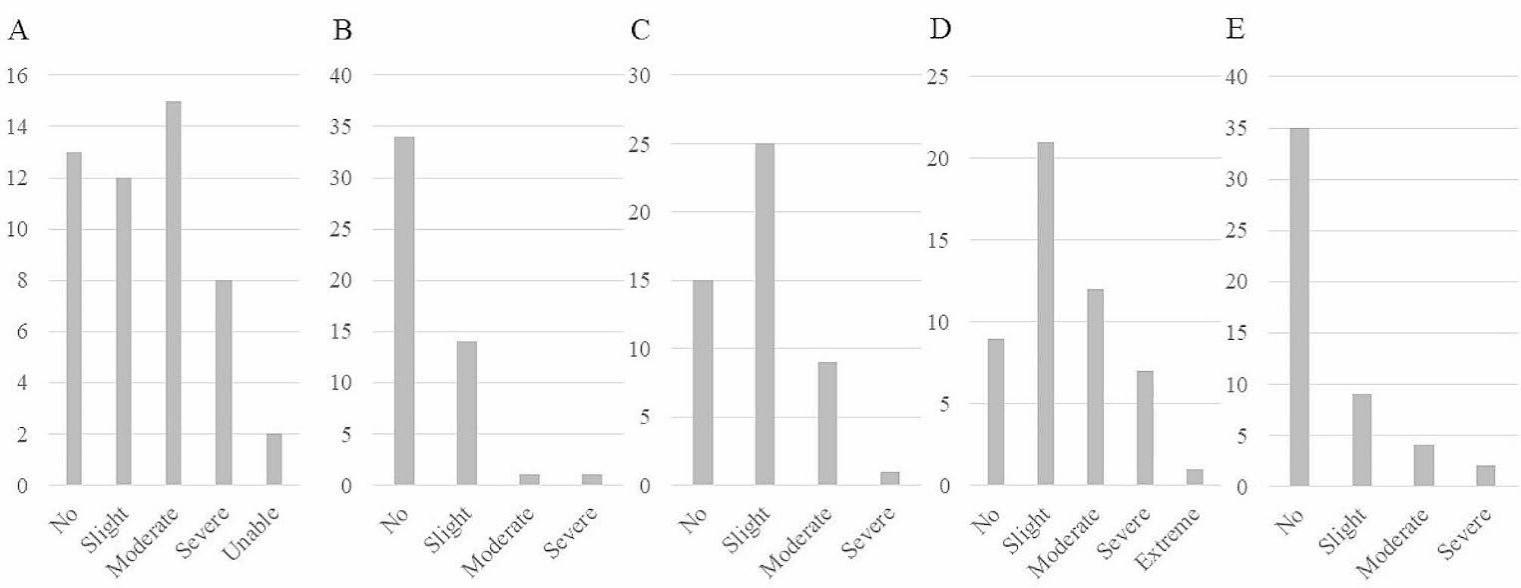
EQ-5D-5 L of all the patients. **(A)** Mobility. **(B)** Self-care. **(C)** Usual activities. **(D)** Pain/discomfort. **(E)** Anxiety/depression

The patient characteristics are shown in Table 1. The disorders were degenerative spinal disorders in 32, osteoarthritis (OA) of the hip in 13, OA of the knee in 4, and other disorders in 2 patients. The background evaluation of QOL by EQ-5D is shown in Fig. 3. The ratio of patients with each domain tends to be high, ranging from slight to moderate, and few patients had severe or extreme disorders.

**Table 1:** Patient characteristics

Number of patients	51
Age	66.1 ± 14.7
	(17–86)
Gender (male/female)	21/30
VAS (mm)	41.3 ± 31.0
EQ-5D-5L Index	0.759 ± 0.169
Cause of disorders	
Degenerative spinal disorders	32
Hip Osteoarthritis	13
Knee Osteoarthritis	4
Others	2
Treatment status	
Before surgery	6
After surgery	28
Conservative treatment	17

### Level of satisfaction and ride comfort

**Fig. 4:**
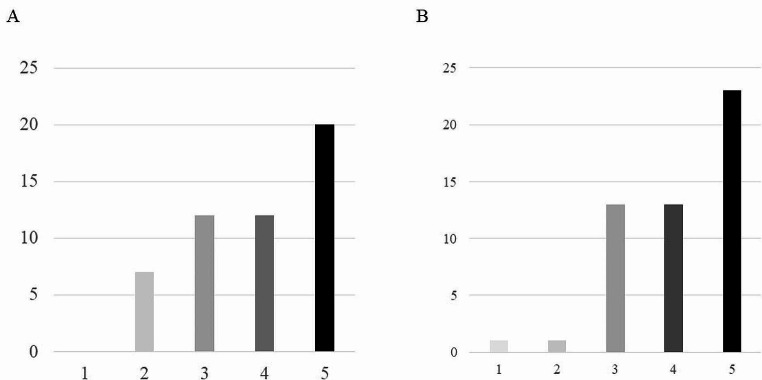
**(A)** Satisfaction of the driverless mobility on a 5-point scale. The highest satisfaction is 5. **(B)** Ride comfort of the driverless mobility on a 5-point scale. The highest comfort is 5

The levels of satisfaction and ride comfort of the driverless mobility on a 5-point scale are shown in Fig. 4. A satisfaction rating of 3 or higher, which indicated generally high satisfaction, was expressed by 44 out of 51 (86%) patients. A generally high ride comfort was indicated by 49 out of 51 (96%) patients who expressed a ride comfort rating of 3 or more. There were no patients who gave a satisfaction rating of 1.

### Trends in satisfaction rating and survey items

We divided the 51 patients into two groups; 32 patients in a satisfaction group who expressed a satisfaction rating of 4 or 5 (Group S) and 19 patients in a dissatisfaction group who expressed a satisfaction rating of 2 or 3 (Group D). The difference between Group S and Group D are shown in Table 2. There were significantly more patients who had already undergone surgery in Group D. The pain VAS score was significantly higher in Group D. Furthermore, the ride comfort rating was significantly lower in Group D. In summary, postoperative patients and those with pain tended to have a decreased satisfaction rating, and those patients felt discomfort when riding this mobility.

**Table 2:** Comparison of demographic and clinical characteristics by satisfaction levels

	Age	Sex		Cause of Disorders				Treatment Status			EQ5D-5L Index	pain VAS	Ride comfort rating				
		Male	Female	Spine	Hip	Knee	Others	Before surgery	After surgery	Conservative		(mm)	5	4	3	2	1
Group S	64.6 ± 15.3	13	19	21	8	2	1	4	13	15	0.760 ± 0.185	34.7 ± 30.1	18	10	4	0	0
Group D	68.6 ± 13.8	8	11	11	5	2	1	2	15	2	0.759 ± 0.143	52.6 ± 30.2	5	3	9	1	1
*P*	0.3438	1.0000		0.9103				0.0187			0.9870	0.0466	0.0137				

### Presence of perceived dangers in the scenes and actual adverse events

In the questionnaire survey after the ride, 26 of 51 (51.0%) patients felt the ride was dangerous. When the mobility passed other patients, 11 felt danger. When the mobility suddenly stopped, 4 felt danger. If the mobility did not move smoothly around people, 2 patients felt the mobility was dangerous. However, there were no actual collisions in any ride. Moreover, there were no other adverse events during the ride.

## Discussion

To our knowledge, this report is the first clinical study to assess the safety and feasibility of in-hospital autonomous transportation services using a driverless mobility for patients with musculoskeletal disorders. While previous studies have explored the satisfaction of healthy individuals with autonomous mobility, focusing primarily on four-wheeled vehicles (satisfaction of driving cars), there is a gap in the literature regarding patient-related outcomes in the context of in-hospital transportation [11]. In addition, although several reports have investigated detailed methods to avoid obstacles such as steps, to our knowledge, there are no reports evaluating patient-related outcomes [12–14]. The present study indicated that the satisfaction levels with this driverless mobility are relatively high. However, postoperative patients and those with pain tended to have less satisfaction than others, and those patients felt discomfort when riding this mobility. The reason for this phenomenon in the present study is presumed to be the inclusion of a significant number of patients with degenerative spinal disorders including failed back syndrome and neuropathic pain due to the spinal cord, which are well-known as intractable pain [15, 16]. Moreover, in patients with osteoarthritis, there were those where pain on the opposite side was progressive after surgery on a unilateral site.

**Fig. 5:**
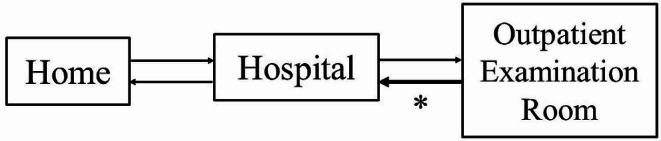
Schema of mobility as a service in medical care. In the present study, we determined the safety and feasibility of travel from an outpatient examination room to the reception area of the hospital (asterisk)

In the present study, there were no severe adverse events such as collisions, and the safety of this driverless mobility was established. Medical Mobility as a Service (MaaS) is a new concept of transportation service that integrates various transportation services as a single service, connecting them seamlessly rather than capturing them as a combination of each transportation service (Fig. 5). In the present study, we determined the safety and feasibility of travel from an outpatient examination room to the reception area of the hospital (Fig. 5, asterisk). Although some problems remain in expanding the service to an on-demand taxi service from a patient’s home to the hospital, the present study can be considered the first step in realizing medical MaaS. By expanding the operation of this driverless mobility system and promoting medical MaaS, it may be possible to develop a reliable, sustainable, and resilient infrastructure that patients with reduced mobility due to musculoskeletal disorders can utilize safely. Moreover, this development holds the potential to ensure that patients with reduced mobility due to musculoskeletal disorders are not left behind by society. That leads to achieving and maintaining sustainable development goals (SDGs) to which orthopaedic surgeons can contribute.

The present study has some limitations. First, the outpatient clinic in our institution is on the ground floor, and we could only evaluate a ride over a straight-line distance and did not investigate the safety and feasibility of entering or exiting of an elevator. However, this seems technically safe and feasible. Further investigation and evaluation are needed. Second, the present study only covered a short period with restricted operating hours. This was primarily due to the restricted availability of the autonomous mobility, which could be accessed free charge only for a limited time. Third, the sample size was small and the patient selection bias existed because the investigation period was limited. A large cohort investigation would be needed to resolve the bias of the cause of disorders. Third, engineering analysis is lacking. However, the main purpose of the present study was to evaluate the patient-reported outcome. A detailed analysis would need to be performed by qualified engineers.

## Conclusions

In-hospital autonomous transportation service using a driverless mobility for patients with musculoskeletal disorders demonstrated high satisfaction levels and was safe with no severe adverse events, such as collisions. Expanding autonomous mobility deployment is expected to achieve mobility as a service in medical care.

## Data Availability

The datasets used during the current study are available from the corresponding author on reasonable request.

## Abbreviations

QOL: quality of life
EQ-5D-5L: EuroQol 5-level
VAS: Visual Analog Scale
OA: osteoarthritis
MaaS: Mobility as a Service
SDGs: sustainable development goals
